# The Hospital. Nursing Section

**Published:** 1903-02-14

**Authors:** 


### The Hospital.

**Nursing Section.**

**Contributions for this Section of "The Hospital" should be addressed to the Editor, "The Hospital" Nursing Section, 28 & 29 Southampton Street, Strand, London, W.C.**

**No. 855.—Vol. XXXIII.** *SATURDAY, FEBRUARY* 14, 1903.

### Notes on News from the Nursing World.

### THE ROYAL VISIT TO THE HERBERT HOSPITAL.

The Royal visit to the Herbert Hospital at Woolwich will take place on Monday next, and the King will accompany the Queen on the occasion. The new quarters for the nurses will be opened by the Queen, and both their Majesties will go through the wards containing the sick and wounded soldiers from South Africa. The Mayor is to present an address of welcome.

### THE WOMEN'S MEMORIAL TO QUEEN VICTORIA.

It seems somewhat late in the day to initiate a movement in Cambridge in support of the Women's Memorial to Queen Victoria. But as it has been decided to keep the fund open until Easter, there is still time to atone for any lost opportunities. The ladies of the University in Cambridge have collected nearly £150, and under the auspices of the Mayor the town ought to be able to contribute a substantial sum. The residents have a special reason for assisting the movement, since it has been agreed that the District Nursing Association is to have 40 per cent. of the total sum collected in the borough. In connection with the Memorial, we offer a suggestion to those who are willing to practice a little self-denial in the season of Lent this year. A material addition might be made to the total already subscribed if some thousands of people denied themselves one luxury every week in Lent and sent the money to the Treasurer of the Memorial Fund.

### ROYAL NATIONAL PENSION FUND FOR NURSES.

The annual meeting of the members of the Royal National Pension Fund will be held on Thursday, March 12th next, at 4 o'clock, at River Plate House, Finsbury Circus, E.C., close to Moorgate Street Station. We give this early intimation of the meeting in order to enable nurses who are policy-holders to at once make a note of the date, so that they may be present at this interesting gathering.

### PERSONAL EXPERIENCE OF NURSING.

In the Bishop of London's speech at the annual meeting of the Hammersmith and Fulham District Nursing Association, which we reported last week, Dr. Ingram referred to his own personal experience of nursing, and said that his recent illness had enabled him to fully appreciate the blessing of a good nurse. This is a compliment to his own nurse, and is one more refutation of the theory that nurses are unpopular. Our own columns afford evidence every week of the increasing popularity of nurses among the poor, and the King, Mr. Chamberlain, and the Bishop of London, have recently illustrated the manner in which their services are valued by the most distinguished.

### THE TRAINING OF GYNÆCOLOGICAL AND MONTHLY NURSES.

The council of the British Gynæcological Society, in view of the fact that at present many nurses undertake gynæcological cases who are not fitted to do so, have determined to at once institute examinations and grant certificates in monthly and gynæcological nursing. Three cardinal points will be enforced, namely, that every nurse certificated by the Society must work only and entirely under the direction of qualified medical practitioners; that no woman will be eligible for examination unless she has had sufficient training both in general and in special nursing; that the Society's certificate will be withdrawn from any nurse who, at any future time, proves to be unworthy of professional trust. The examinations will be held quarterly, and full particulars can be obtained from the hon. secretaries, 20 Hanover Square, London, W.

### AN ABUSE OF UNIFORM.

We hear that the matron of a workhouse in the north of England has put her female servants into nurses' uniform, including cap strings. Her action is the more reprehensible because she is herself a trained nurse, and we do not wonder that the charge nurses of the union infirmary are indignant at her conduct. She is not the first offender of the kind, but her abuse of uniform proves that training does not always suffice to endow a woman with a sense of the fitness of things.

### PRIVATE NURSING IN CAPE COLONY.

Although, as we have stated, the supply of nurses in some parts of South Africa is in excess of the demand, this is not, we understand, the case in such towns as East London and Queenstown in Cape Colony. Here private nurses are badly wanted, especially if they have had maternity training; while in some of the smaller towns it is often quite impossible to get a nurse of any kind. It is, however, necessary to emphasise the fact that the conditions of nursing are extremely unlike those at home. The nurse makes herself one of the family and must be ready to turn her hand to anything. A knowledge of cooking is requisite, if not indispensable. The fees are generally good, but vary very much in different places. It may be added that though life in these towns has many attractions for any who can adapt themselves to the customs of the country, most of the comforts and conveniences enjoyed in England are lacking.

### THE NURSES' DANCE AT EDINBURGH.

The nurses' dance given by the Lord Provost of Edinburgh, the Convener of the Public Health

Committee, and the Town Council has grown from a very small affair to a great popular function. It was started as an "At Home" in the Council Chambers, but this year the accommodation afforded by the music hall and assembly rooms hardly proved sufficient. Out of a total of nine hundred guests, there were upwards of four hundred nurses present, including representatives from most of the important institutions. An artistic river scene hid the organ in the music hall, and the decorations generally were very effective.

### STROUD NURSING ASSOCIATION AND THE PENSION FUND.

At the annual meeting of the Stroud and District Nursing Association last year it was intimated that the committee had decided that every nurse on the staff who joins the Royal National Pension Fund for Nurses to ensure a pension of £15 payable at the age of 50 should be assisted by the Association repaying them at the rate of 25 to 50 per cent. on each year's premium according to the length of service. In the report for 1902, which was read and adopted at the annual meeting last week, it was announced that four of the nurses had availed themselves of the offer made by the Association. This is encouraging, and should have the effect of prompting other nursing organisations to offer a similar advantage to members of their staff. The financial statement shows that the expenses of the Association amounted to £913, and that the balance to the good of £19 has been reduced by a trifle. The record of the work of the nurses is excellent, but more nurses are still required and will be engaged in districts round Stroud which are at present without them as soon as the funds will permit.

### THE BATH RESOLUTION.

The resolution passed by the Bath Board of Guardians suggesting as an alternative to the creation of "qualified nurses" the adoption of the term of "qualified probationers" to nurses with one year's training in nurses' Poor Law schools has been approved by the Wellington, Loughborough, Canterbury, and Bury Boards of Guardians. The Worcester, Hartismere, and Alnwick Boards have passed resolutions to the effect that the communication "lie on the table." At a meeting of the Richmond (Surrey) Board of Guardians the matter came up for discussion when Mr. F. W. Dimbleby strongly opposed it, urging that as they had no resident medical officer, and were a minor training school, they would be making their nurses only "qualified probationers" instead of "qualified nurses." It would put them at a very great disadvantage if they left the Poor Law service. No action was taken in the matter. The Builth and Bingham Boards are among others which have also determined not to take any action.

### THE OPPORTUNITY OF NORTHAMPTONSHIRE.

Admirable speeches were delivered by Lady Knightley and her cousin Lady Knightley of Fawsley at a meeting held in Towcester the other day in support of the proposed scheme for a County Nursing Association for Northamptonshire. Lady Knightley expressed herself strongly opposed to the employment of imperfectly trained nurses, and asked why the county should go to the trouble and expense of training persons when fully trained and qualified nurses could be obtained without difficulty. If, as Lady Knightley of Fawsley pointed out, the committee of the Daventry Nursing Association can make two ends meet by means of subscriptions and the pence of the poor, why should an organisation be started under more imposing auspices with a staff of imperfectly trained nurses? We do not agree with the view that the village nurses described by Miss Hughes are worse than none at all. But the fact that the Daventry Association, started six or seven years ago, in two parishes, with plenty of cold water thrown on it, has been extended to 22 parishes, and employs four fully trained nurses, should encourage the promoters of a county organisation to commence on the same basis. It is still not too late to aim high; for at Towcester the only resolution passed was that the meeting would give the movement "its best consideration." We hope that the Northamptonshire people, who have plenty of enterprise, will, as the result of the further consideration to which they are pledged, decide to establish an association on as sound and satisfactory a basis as that of Daventry.

### ASYLUM NURSES AND THE COLNEY HATCH FIRE.

At the Colney Hatch inquest held last week several of the nurses of the asylum gave evidence. Nurse Hayling, night nurse of dormitories X 3 and X 4, appeared with her right hand in bandages, the result of burns. She said that as soon as she was told of the fire she went and roused the night nurses in other dormitories, and helped to get the patients into the corridor. As fast as some of them were brought out of the dormitory they ran back. Nurse Ethel Lambert, a day nurse in X 5 ward, described a terrible struggle she had with one of the patients she attempted to rescue, who nearly succeeded in throwing her into the burning cupboard. She had only her nightdress and a pair of slippers on. Nurse Ada Grover said that during 12 years' experience of the asylum she had never known matches to be used, nor had she seen any nurses smoking cigarettes. Nurse Walford remained with her patients in X 4 ward, although it was full of smoke, broke the glass box which contained the patent key of the door leading into the grounds, and got out her patients all in their night attire. Miss Alice Greenaway, the head night attendant, gave her evidence under difficulties as she had lost her voice from the effects of the fire. She had a key to open the door into the grounds, but the patients fought so desperately that she dropped it and it could not be found again. The nurses could not open the doors leading to the grounds.

### A SUCCESSFUL NURSING INSTITUTION.

At the twenty-seventh annual meeting of the Leeds Trained Nurses' Institution, it was stated that the staff now number 86 members engaged in private nursing, and 21 probationers in hospitals. The increasing demand for the nurses is shown by the fact that 169 cases had to be declined during the year in consequence of the nurse being engaged when application was made. Yet the number of cases attended was 3,202, and the number of visits paid 60,032. No less than 19 districts are covered by the organisation. We note that the sum of £250

was paid by the Institution to the Leeds Trained Nurses' Trust Fund of the Royal National Pension Fund for Nurses.

### THE ANNUAL REPORT OF ST. PATRICK S NURSES' HOME

Last week the annual meeting in connection with St. Patrick's Nurses' Home was held in Dublin. The Archbishop of Dublin presided, and there was a large attendance of the supporters of the institution. On a previous occasion, as we mentioned in our last issue, his Grace preached on behalf of the institution, and at the meeting he spoke in the warmest terms of its work in the sister island. Portions of the report were then read by the hon. secretary, Sir Edmund T. Bewley. The home has now been in existence for 26 years, and during the past twelve months the number of cases were 2,100, while the number of visits paid reached the very large total of 39,788, being an increase in the former, as compared with 1901, of 240, and in the latter of 2,020. A number of the cases were in the wealthiest and most thriving suburbs; and, on the other hand, the entire services of two nurses and half the time of a third were devoted to nursing the poor in Rathmines and Rathgar. The development in the work, which twelve years ago was limited to 534 cases and 11,138 visits, has necessarily been accompanied by an increase in the staff, which now consists of the superintendent, the district superintendent, seven nurses, and five Queen's probationers. Last year ten probationers were trained in the home, and another gratifying event in its history was the gift of £1,100 from an anonymous donor. We agree with one of the speakers at the meeting, that the state of affairs indicated in the report reflects the greatest credit on Miss Howell, the superintendent, and Miss Berry, the district superintendent, who is largely responsible for the training of the probationers.

### A COTTAGE HOSPITAL APPOINTMENT.

The Governors of the Westminster Memorial Cottage Hospital at Shaftesbury have very wisely appointed as matron a highly trained nurse. Miss Jane Ord, who will enter upon her duties on the 25th of next month, and act both as matron and nurse, was not only trained at Guy's Hospital for three years, but was on the private nursing staff for some time. She has since been sister at a general hospital, matron of an ophthalmic hospital, and temporary matron of a children's hospital. There is no doubt that the matron of a cottage hospital, who has to do all the nursing herself, should, if possible, have enjoyed this kind of varied experience which, at any moment may be of practical use to her.

### THE RESIGNATION OF MISS NEWBURY.

The strain of the arduous duties at Dudley Workhouse Infirmary, accentuated by difficulties not of her own creation, has compelled Miss Newbury to resign her appointment as superintendent nurse. We suppose that the Guardians who have been complaining of her ever since she refused to give up her office at their behest, after being justified in her action by the Local Government Board, are now satisfied, though it remains to be seen whether her successor will prove any more acceptable. Miss Newbury is, of course, to receive a testimonial, but it is a great pity that she should have virtually been driven to relinquish a post for which she has shown she has the necessary strength of character. We hope that her next appointment will be free from the annoyances which seems to have been her portion at Dudley ever since she endeavoured to introduce reforms.

### A HOT-WATER BOTTLE CASE.

At the Manchester Assizes last week Mr. and Mrs. Kelsall brought an action against Thomas and Mary Ann Hacking to recover damages for personal injuries. The defendants counter-claimed for £63 for the services of a nurse. It appeared from the speech of the counsel for the plaintiff that four years ago Mrs. Kelsall had to undergo a serious operation, and her husband sent to the medical and surgical home conducted by Mr. and Mrs. Hacking for a trained nurse. On that occasion the operation was most successfully performed, and two or three weeks after it Mrs. Kelsall was up and doing well. In July, 1901 it was found necessary that a second operation should be performed, and Mr. Kelsall again sent to the home for a nurse. The nurse who formerly attended his wife was not available, and another was substituted. The plaintiff's case was that owing to the negligence of the latter in applying a hot-water bottle to Mrs. Kelsall's leg she sustained a very serious injury. The defendants denied either negligence or want of skill on the part of the nurse, and claimed payment for her services. But before the defendants were heard, their counsel announced that "in view of the pain and suffering which Mrs. Kelsall had undoubtedly experienced, be the fault whose it might, his clients would accept judgment for the plaintiff for £126 and costs, and abandon their claim for the services of a nurse." It is obvious that the plaintiffs' case must have been unanswerable, and whoever was to blame, the result was as disastrous financially to the defendants as it seems to have been physically painful to Mrs. Kelsall.

### THE PRIVATE NURSES OF SWANSEA HOSPITAL.

The Swansea Hospital Board of Management have now organised their private nursing staff. The department is to be managed by a sub-committee of five members. Probationers admitted to the hospital are to be in training for three years, and it will be optional for them to remain a fourth year as a member of the private nursing staff. The rate of payment adopted is: first year, £30 and 5 per cent. of earnings; second year, £32 10s. and 7½ per cent. of earnings; third year, £35 and 10 per cent. of earnings; fourth year, £37 10s. and 10 per cent. of earnings; fifth year, £40 and 10 per cent. of earnings, the last being the maximum. The charges are to be: for one week or part of a week for infectious diseases, £2 2s.; for one day or one night, 5s.; attendance at an operation, 7s. 6d.; if the nurse sleeps at the hospital, 10s. 6d. per week extra; laundry and travelling expenses extra. The hospital is to be charged the same fees as private patients when the services of the private nurses are required in the wards; and the private nursing department is to be charged 3s. per day for the maintenance of each nurse when not employed.

### SHORT ITEMS

The s.s. *Orbona*, which arrived at Southampton last week, had on board Sister L. L. Holmes, of the Army Nursing Service Reserve.

### The Nursing Outlook.

> " From magnanimity, all fear above;
> From nobler recompense, above applause;
> Which owes to man's short outlook all its charm."

### THE NURSES' CO-OPERATION.

THE annual meeting of the nurses of the Co-operation was held last Friday, and the annual meeting of the members last Tuesday, when the twelfth report was presented and passed. The Co-operation is a society, registered under the Board of Trade, for the purpose of securing to private nurses their own earnings, with merely the deduction of a small percentage for office purposes. It has been very successful throughout its career, and now has close on 500 nurses working for it, with a turnover of £46,000 a year. It has been copied in its objects and methods by many other groups of nurses, and is a fine example of what women can do when they really try to pull together. But there have been signs during the last few years that the pioneer spirit of its founders does not exist amongst the new members, and that unless the nurses develop public spirit and courage, the Co-operation will become like unto the old restricted proprietary establishments, and the nurses will lose their freedom and their fees.

For instance, only some 45 nurses attended their meeting, and though there were several points which nurses wanted raised no one brought them forward. Again, in voting for the nurse-representatives 517 papers were sent out, and only 236 were filled in and returned. Surely this shows on the part of the nurses a lack of interest in their own affairs, which may easily be interpreted into an evidence of satisfaction with the existing management. Silent discontent and private criticism are useless, and it behoves the nurses to choose their representatives carefully, instruct them fully, and see that they press forward the wishes and opinions of those whom they represent. On this the future success of the Co-operation will largely depend. The nurses elected are Theresa Thorpe, Mary Tonks, Annie Austin, and Hallett Baker. We hope that these ladies will fully realise their responsibilities and insist on their views being heard and respected. It is always difficult for new representatives to hold their own against the serried ranks of officialdom, but unless the elected representative nurses are prepared to do this when necessary, they are false to those who elected them. We should like to impress on them and on all nurses, that it was the public spirit and patient devotion of their own ranks that formed the Co-operation, and their duty to the founders and to the nurses' cause is to carry it on in a similar spirit.

At the members' meeting Mr. Pitts and Dr. Turney were re-elected on to the committee, and Mrs. Makins and Mr. Cuthbert S. Wallace were put on to fill the two vacancies. The last election shows how largely the Co-operation is in the hands of a single hospital to which all those members belong. Moreover, Mr. Wallace was only elected a member in December last, and already he is elected to the committee. No wonder that it is believed in medical circles that the Nurses' Co-operation " belongs to St. Thomas's " !

The agenda and report are as meagre as possible, and the nurses can glean but little information regarding their own society from the printed matter given them. All the points called for by the nurses at their meetings of protest last year are ignored: details of salary are not given; the same secretary and treasurer remain, though the nurses asked for their resignation. The Home is again run at a deficit, and £750 is taken from the General Fund towards paying off the loan on the Home.

It is, of course, very difficult to get nurses to understand all that underlies these points. Only one side is ever put before them at their meetings, and only certain members of the committee ever attend these meetings, while other members are never even informed that they are coming off. We understand that so little do the nurses know of this that they in all innocence wrote to four of the old members asking them to attend the meeting on February 6th and criticise the accounts and the report. In the early days of the Society, before the management was captured by a clique, there was always a public meeting once a year to which the members, nurses, public, and Press were alike invited, and at which some celebrated person—Sir William Broadbent, for instance—was asked to take the chair. So keenly do many nurses resent the present policy that there is a movement amongst them to hold a public meeting entirely on their own account, and by money subscribed amongst themselves, and to put forward at the meeting certain speakers to explain their views. The nurses themselves prefer not to speak in public for fear of raising a prejudice against them in the minds of the authorities. It seems to us a pity the nurses should be put to this expense, for if only they would all stand by one another it would be quite easy to force the committee to come forth into the light of day once more, and let the nurses know all that is going on. If the new nurse-representatives are what they ought to be, they might at least get this matter of freedom of meeting between the nurses and all the members put right. We would refer anyone who wants to understand the question of the Co-operation quarrel to an article by Miss Kimber in " The Transactions of the Third International Congress of Nurses." Miss Kimber is apparently an American nurse who came over here to study new movements, and her astonishment at the way the nurses have allowed a powerful organisation of their own to be controlled by a few people who are not nurses is an eye-opener to those who think only of fees and a quiet life, and are contented to be domineered over so long as they get their dinner regularly.

In conclusion, let us admit that committee work is hard and thankless. We do not wish to ignore the work or depreciate the motives of those who undertake it. But unless all the members of the committee really represent the whole body of the nurses they have no reason for existence. The Co-operation belongs to the nurses; that is the principle at stake. Let the nurses realise this and act upon it. Whenever they forget it there is a danger that the organisation will fall under the control of a small but self-assertive coterie of persons who are not nurses, but private individuals with leisure and ambition to force their control and discipline upon a body of women workers who are worthy of a better fate.

### Lectures on Medical and Surgical Nursing.

By John Hopkins, F.R.C.S., Medical Superintendent of the Central London Sick Asylum District Asylums, Cleveland Street, W., and Hendon, N.W.

### LECTURE I.—MICROBES.

Having learned something of the structure and functions of the body, you are now in a position to carry your studies further, and to learn what are those variations from normal health that we call disease. The temperature of the body in health is as you know 98·4° Fahr. When, by the use of the thermometer, you discover that it has risen above this point you know that there is generally an inflammatory condition somewhere in the body. Too much heat is being produced. Now one of the commonest causes of excessive heat production is the growth of micro-organisms. So common are they as a cause of disease that it becomes your duty to learn something of their nature as they grow outside the body, in order that you may the better understand their growth, when in the blood or tissues. What is an organism? It is a living thing; it may be a plant or an animal. We see around us huge trees and a great variety of living plants, some of which are very small, as, for example, the mould which grows upon a pot of jam: animals, too, of great variety of size and shape, the smallest of which are just visible to the naked eye. But these are not all; for when we use a microscope, it is discovered that there is a world of animals and plants only to be seen under a magnifying glass. These are all micro-organisms. Many of these do not cause disease in the human body, and with them we have very little concern. Those that do, however, are very numerous, and need further attention. All living things are organised. The word organisation originally meant the possession of organs, such as heart and stomach. But now it signifies things that have or have had life, however deficient in organs they may be. The simplest living animal is the amœba. It consists to all appearances of a minute drop of jelly. This drop of jelly crawls about by altering its shape, thrusting out one side and drawing in the opposite one, takes food and grows, by clinging to particles that come in contact with it and slipping them into its interior at any point touched. It breeds by two amœbæ coming into contact and flowing together to make one being, and then presently by dividing up into many young amœbæ, each of which starts off on an independent course. To all appearance quite structureless, we nevertheless call this an organism, for it lives, produces young, and dies. The amœba is the simplest type of animal life, and our knowlege of animal structure is based upon it. It is a single cell. Place several cells together and you have the first step towards a better organisation. Many of the lowest forms of animal life are only a mass of amœbæ-like cells lying together, each cell dependent upon the rest for a basis of support, but living otherwise independently. Take a little higher step in organisation and you will find that the cells do not all perform similar functions. There is a division of labour, some performing one task, and some another for one common end of all. This appearance of a difference in the duties performed is called the process of differentiation. In the next step upwards it may now be found that the mass of cells is formed into a bag-like organism, and that the cells lining the bag are concerned in digesting what particles fall into it, whilst those on the outside are concerned merely in gathering particles to put in the bag, the outermost layer acting as organs of touch, determining what to take and what to reject.

With the differentiation of function comes a difference of structure, the cells changing their shape and arrangement to meet their special calling, those that are principally concerned in movement taking the form of muscle cells. The next advance is the beginning of the formation of a nervous system, a process extending from a superficial cell down into the mass of muscle cells with which it comes into close contact, so that when the outer cell is touched by an object the muscle is set in motion. Thus by degrees all the tissues of the body are formed from the primitive amœboid cells of which any animal organism is at first composed.

The growth of a tissue is brought about by division of the primitive cells, whereby they may be multiplied as much as need be.

On turning to a study of plant structure we find the same principle applied. By the adhesion of cells and by their differentiation there is a progressive organisation in their growth, multiplication of cells by division being the means of that growth.

Turning now to the micro-organisms which more especially concern us, we find they absorb nutriment, multiply by division and die. They vary much in size, have different shapes, remain in some cases single like the amœba, and in others again, several adhere together in chains, or in masses, or in other ways. According to their kind they live, some upon vegetable matter only, some upon animal matter only, and yet others live upon both vegetable and animal.

These microbes exist in very great abundance and multiply with great rapidity. Wherever there is dust they are to be found. They are very minute, the smallest of them known needing a very high power of the microscope to show them as mere dots or rods. The dots are called cocci, when they form chains they are called streptococci, and when masses, staphylococci. The rods are called bacilli. Some are corkscrew shaped, and are known as spirilla. When decay of animal or vegetable matter takes place, it is due to the growth of microbes. When food goes bad, it is the growth of micro-organisms that causes it. They cause milk to turn sour, butter to become rancid, broth and beef tea to turn. Swallowed they cause vomiting and diarrhœa. In a wound they cause inflammation and the formation of pus. In the tissues they excite inflammation, abscess, septicæmia, and pyæmia, besides inflammation of the organs of the body and fevers.

It is astonishing how rapidly these lowly organisms increase and multiply. Think for a moment of the difference there is in the rapidity of reproduction of different animals. Young may be produced one at a time once in two or three years. On the other hand, one litter of young may succeed another every few weeks. Fish are often very prolific. The roe of the herring is a mass of eggs some thousands in number. The growth of the yeast plant is so rapid that its individual cells can be seen under the microscope to spring up as buds upon the parent cell and to grow to full size; these presently to give off buds themselves that expand in like manner. In the case of the yeast plant the cells remain attached to each other and thus form branches of elongated cells fixed end to end. In other cases the buds drop off, so that the plant never takes any definite shape, but remains as a mass of free cells. If a new cell be formed every minute by each of the cells present, you may calculate the number that will be produced in an hour. Thus at the end of the first minute there will be two, in two minutes, 4; in three minutes, 8; and so on. In five minutes there will be 32, in 10 minutes the number will have increased to 1,024, and in 15 minutes there will be 32,768 cells, the progeny of the single cell which existed a quarter of an hour before. By this you may judge of the rapidity of the growth and multiplication of micro-organisms.

Now, if a microbe be allowed to grow upon a little gelatine, it is sure to increase and multiply with great rapidity, and presently it forms a speck of mould visible to the naked eye, and the gelatine is so changed that it may liquefy and give off a bad odour. The microbe has been living upon the gelatine, decomposing it, absorbing nutriment from it and excreting, may be, its own waste products.

The same thing happens when a microbe gains entry into the living body. It grows and multiplies, living upon the juices of the tissues. It is the action of the living microbes and of their waste products upon the blood and tissues, which gives rise to the phenomena of inflammation, that will be the subject of the next lecture.

### After Fifty: Or Poor Law Nurses and Pensions.

By Miss WILSON, Treasurer of the Workhouse Infirmary Nursing Association; Member of the Midwives' Board.

(*Continued from page* 264)

### The Royal National Pension Fund.

I now come to the second argument, with which my sympathy is very strong—*i.e.* that the present Pension and Superannuation Funds do not fairly meet the difficulties of Poor Law nurses. I will consider the Pension Fund first, strictly in relation to Poor Law nurses. Its success as a fund for the benefit of nurses in general can only be observed with increasing admiration. I had been for some time engaged in Poor Law work, and had seen some very sad cases of nurses broken in health, often from overwork, with no form of help, some years before the Fund was formed. Of the opposition it met with and its history, militant and triumphant, I have been a close observer, for I knew only too well how urgently its work was needed. The committee of the Workhouse Nursing Association have, in and out of season, pressed the advantages the Fund offers upon the nurses who at varying times worked under our care, and I am glad to say that a fair proportion have joined. Some are now reaping the benefit of their wisdom, partly because we have placed the needs of the future often so urgently before them, partly because they themselves were able to see the exceptional kindness and individual care taken by the officers in each case; how, for example, a delay in paying premiums would receive careful attention at the office, and how fairly policy-holders were treated when a question of ill-health or family claims arose. I can only "testify that I have seen," and I would add that for patient kindness, for tact, for truly fulfilling the work in the spirit in which it was begun, the Royal National Pension Fund takes a high place. In many metropolitan infirmaries the advantages of the Superannuation Fund are constantly placed before nurses; if it were possible to do this in regard to the Pension Fund, and could its method of payment be simplified by the collection of premiums, I think the figures quoted regarding the two funds might be materially altered.

### Why Nurses Fail to Join the Fund.

But when all has been said, how disappointing are the figures I have quoted. Here is a well administered fund, here is an ever-increasing army of Poor Law nurses, and how slight is the approximate touch of these two bodies with each other! Many and various are the reasons given; that the Pension Fund is too expensive a bank for the ordinary infirmary nurse is, I fear, one of the chief obstacles. Salaries, after training under the Poor Law, average £25 to £35 for sisters; for superintendent nurses from £35 to £50 (I am not dealing with the position of matrons or assistant matrons). With these salaries a nurse must have firm principles and clear foresight to put aside annually what is to her a considerable part of her earnings. It would be too much to say that she must be a remarkable person to do so, but she certainly must be a woman with strong self-respect, with decided views on the duty of self-help, and one who is not lightly influenced by every wind of doctrine, either as regards her own "right" to enjoy life, or the superior advantages of the building society in her native town, in which she is told her brother-in-law "is making a fortune," or the "certainty" that many nurses will be needed in South Africa and the consequent wisdom of drawing out her premiums and taking her passage forthwith. While in short she should have common sense, she need not be pictured wholly as a person who must "scorn delights and live laborious days." Nevertheless, while the action of the woman engaged as a private nurse, earning £80 clear, who takes advantage of the Pension Fund, is sensible and calls for no undue self-denial, the infirmary nurse earning at the rate of £30 per annum who takes out a policy in the Pension Fund shows a distinct fibre of character.

### Other Reasons.

An authority on the subject says:—"I am convinced that a very much larger proportion would save if they could only be induced to take the first step of entering for the minimum annuity, and later on increasing the amount assured, as their position improves. It is the old story of 'what is the use of £15 a year? I would rather starve.' One cannot induce the majority of nurses to appreciate the half-loaf principle." It is so much easier to talk at 24 of one's willingness to starve, than to face the actual fact at 60. It should be noted, however, that friends and relations are much more ready to help when there is a small annuity assured; the burden of complete support is otherwise too onerous to be undertaken. Matrons of infirmaries might do a great deal by pointing out to young nurses the desirability of making some provision for the future. It is, of course, natural that matrons should feel some reserve about interfering in what is, after all, the private concern of the nurse, but the matter is so important that it would be well if they could overcome this reluctance and would use their undoubted influence in the clear interests of those who are under them. Young women seldom see for themselves the advantage of saving, whereas a word in season from a respected matron might induce them to give serious consideration to a subject which so vitally affects their future.

There is one point that probably makes joining the Superannuation Fund an easier process to nurses than becoming a member of the Pension Fund, viz., in the case of the latter Fund a nurse has handled her money before she parts with it, while under the Superannuation Act a deduction is made before she receives her salary. We all know how much less acutely the majority of people feel a deduction from income than the actual payment of the amount in cash, apart from the minor worries of letter writing, obtaining postal orders, etc. It may be urged that these are very trifling matters, but in a busy life, little duties make a great difference, and the way of least resistance is the easiest path to follow.

### AFTER FIFTY: OR POOR LAW NURSES AND PENSIONS—*Continued.*

### The Payment of Half Premiums.

If Boards of Guardians acted as hospitals do in a few cases, and paid half the premiums of nurses who continue in their service for a certain number of years, the matter might wear a different aspect; but it is questionable if the ratepayers would consent to such an arrangement while the Superannuation Act applies to nurses; it must be noted also that the number even of hospitals who pay half the nurse's premium is disappointingly small. The premiums of the Pension Fund cannot be made lower with actuarial safety, but they appear to be proportionately high to a large number of Poor Law nurses whose salaries cannot in the nature of things be materially raised in the future. Salaries under the Poor Law are now as a rule quite as high as those of nurses working in hospitals, and therefore the remedy for the present state of things is scarcely to be found in any immediate hope of increased pay.

I would advise any nurse who desires to join the Pension Fund to arrange for an interview with the Secretary, at the offices, 28 Finsbury Pavement, where she can obtain clear and exact information of its many advantages.

Nor do I think that much relief would be effected by the adoption of Dr. Solomon Smith's suggestion made in his interesting series of papers published in The Hospital some time ago, that educated women of 40 years and upwards who were weary of the "rush" of hospital and private work, might be induced to take posts as superintendent nurses in country infirmaries: the question of pension naturally creates a difficulty to this scheme. There is no doubt that, in view of the fact that under the Superannuation Act it is the last union—from which she finally retires—that has to pay a nurse's pension, guardians quite rightly have the fear of the ratepayers before their eyes, and are shy of appointing nurses who are not in the prime of life.

*(To be Continued.)*

### A Visit to the Santa Maria Nuova Ospedale, Firenze.

BY AN OCCASIONAL CORRESPONDENT.

The Santa Maria Nuova Ospedale occupies a very central position in Florence; it is near the Duomo and Baptistery, and as the tram service is exceptionally good, is within easy reach of all parts of the city. It contains about 600 beds and is the largest hospital in Florence. Very familiar to the Florentines is the spectacle of a white-robed procession, the Brothers of the "Societá della Misericórdia" bearing, with masked faces and with swift and echoing footsteps, an ambulance stretcher to the friendly doors of this great institution where succour will be afforded and, if possible, health restored. The wards are lofty and well ventilated, the walls are painted in a light shade of grey, and the floors are paved with deep red Dutch tiles.

### The Helpers and Assistants.

Each ward contains about 26 beds and is under the charge of a Sister of Charity, who is assisted by two female helpers in the female wards, and by male assistants in the male wards. The status of these assistants is difficult to define. They are neither wardmaids nor probationers, but perform the duties of both; in fact they do all the menial work which is beneath the dignity of "la religieuse," but never rise to any position of responsibility in the hospital. The female assistant wears a long white garment with sleeves, which very much resembles a French pinafore, and no cap upon her hair, which, I regretted to observe, was not always in perfect order; while the male helper wears the long cotton blouse so universal on the Continent, varying in colour, but always of the same pattern. The doctors, while in the hospital, wear long white coats; surely an excellent arrangement.

### No Resident Doctors.

The young medico who was commissioned by the director to show me round nervously inquired of the porter if I could speak Italian, whereupon I hastened to set his mind at rest, and as soon as he was satisfied that no English would be required of him, he proceeded most courteously to give me all the information I desired. In answer to my inquiries, I was told that no doctors reside in the hospital, but a certain number are always on duty during the day, and always two at night. There are three grades of medicos, which seem to be equivalent to a senior house surgeon, a junior house surgeon, and a medical student; only as they take turns to be on duty there are a large number of them. A junior always accompanies a senior medical officer when making his rounds through the wards. There are two large operation rooms, one for aseptic, and the other for septic cases. I observed that the latest automatic arrangement for washing hands was provided—that of pressing a brass pedal on the floor—to cause water to flow into the basin above, thereby avoiding the possibility of infecting the fingers by turning a tap during an operation. The instruments are kept in an ante-room, and are cleaned and kept bright by the "Sister" herself. A large ward is set apart for the reception of new cases, where they remain for a few days until the doctor is satisfied that they are not suffering from any infectious disease.

### The Private Wards.

I inquired for the children's ward, but was told that the hospital only admitted adults, and that no special cases, such as infectious fevers, skin diseases, etc., were treated there. A particular feature of the hospital is the provision of private wards for paying patients, with payment as follows: first-class single wards, ten francs per day; second-class double-bedded rooms, six francs per day. These wards are very comfortable, and look most inviting. Each one had a stove in it. There is also a large, pleasant sitting-room for the use of convalescent patients and a separate operation-room. These wards were in charge of a bright-faced "Sister" who accompanied me round her domain with evident pride.

### A Good Custom.

Below on the balconies the convalescents were walking about and enjoying a quiet smoke. A most excellent plan prevails in Italy which I could not refrain from wishing might be adopted in England. Every male patient on admission is supplied with a suit of white linen, including a cap. The suit is worn all the time he remains in the hospital, a clean one being supplied periodically, and the patient's own clothes are stoved if necessary; otherwise they are taken to a store-room and given back when he is discharged. Large and convenient bath-rooms bear witness to the fact that at any rate while in hospital, the Italian is required to submit to a bath; and the sanitary arrangements are excellent.

### The Nursing System.

One cannot visit the great Italian hospitals without realising that considerable improvements have taken place during the last few decades in the surroundings and treatment of the patients. Of course, to the eyes of an English nurse, accustomed to the order and discipline of an English hospital, the nursing system leaves much to be desired, but as long as such a strong prejudice exists in Italy against nursing as a profession for ladies, so long will the doctors be compelled to employ the services of religious sisters as "heads of wards." One other factor to be considered is this, that the hospitals are in some cases supported by "religious societies" and certainly they are under strong Roman Catholic influence, so that it would be very difficult to alter the present state of things. But doubtless the changes which have taken place in many of the French hospitals will, in course of time, be introduced into Italy.

### Everybody's Opinion.

**[Correspondence on all subjects is invited, but we cannot in any way be responsible for the opinions expressed by our correspondents. No communication can be entertained if the name and address of the correspondent are not given as a guarantee of good faith, but not necessarily for publication. All correspondents should write on one side of the paper only.]**

### "QUALIFIED" NURSE AND POOR LAW INFIRMARY MATRONS.

"H." writes: While the question of creating a third grade, viz., "qualified" nurse, in Poor Law nursing is occupying a good deal of attention, I should like to recall to the minds of the public interested the fact that it is largely due to the far-sighted ability and perseverance of a few of the influential workhouse infirmary matrons, aided by intelligent committees and some outside philanthropic people, that Poor Law infirmary nursing has been placed in the front as it now is. There is, therefore, no fear of these going back They have worked hard to bring things up to a high standard, and have spread abroad their views with the people they have trained and helped to place in good positions. So we need not anticipate that the half-trained nurse will be tolerated or has "come to stay."

### COST OF MAINTAINING A BICYCLE.

"B. M." writes: In reply to "Cyclist" I should like to say that if a bicycle costs 10s. quarterly to keep it in repair the bicycle must be on its last legs, or rather on its last wheels, or the tyres are tired out with district work. I am a district nurse, and do a fair amount of cycling, and my bicycle has cost not more than 25s. for the whole of last year. That amount includes new tyres, so that I do not expect to be at much expense for repairs in the coming year, unless, of course, I have some serious accident. If "Cyclist" had a good bicycle to begin with, 10s. would be ample for repairs yearly.

### NURSES AT CONSUMPTION SANATORIA.

"OBSERVER" writes: Your correspondent, "One Who Wishes to Know," raises two or three questions of unequal importance. All table utensils in a sanatorium for consumptives should be properly cleansed, not merely "roughly rinsed," and the matron or housekeeper should see that this is done. Washed properly in the usual way, and dipped into clean hot water, such articles are most unlikely to convey infection, as they have a glazed or polished surface and smooth edges. Whether the nurses in the sanatorium have a separate set of utensils is entirely a matter of convenience. As a rule the dinner service is of the same pattern; but there is often a separate tea service. Excreta used as manure are unlikely in this country to be dangerous, provided that the vegetables produced are not eaten raw. Vegetables cannot be grown on fresh excreta; not, in fact, until the latter have been completely transformed into earth, and the tubercle bacilli are presumably dead. Even if any bacilli were still alive, they would never survive the cooking process.

"P. B." writes: I think it is a good thing that "One Who Wishes to Know" should write for information, as her ignorance concerning open-air sanatoria might alarm those who do not know how carefully most of them are managed. I have had some experience in one as a nurse, and should like to draw the attention of "One Who Wishes to Know" that were she to be initiated into the workings of a large sewage farm she would probably find that the Covent Garden sprout was reared on the excreta of some large general hospital. At the sanatorium I was at we also used the same crockery as the patients, but had the privilege of having it washed up first, and as this was done very thoroughly there (the things are put into a sink and boiling water poured on to them, in which they remain some time before being finally cleaned), we had no fear of infection. It may not be generally known that the patients in a sanatorium are not allowed to leave any scraps of their food, so that no one's health can be endangered by partaking of what their forks have touched. If these precautions were not taken what would become of the patients slightly affected who go to be cured? We ate the same food that was prepared for the patients, and this was probably the cause of the alarming increase of weight from which most of the nurses suffered.

"MRS. M. O. SINCLAIR, Lady Superintendent of Rostrevor Sanatorium, near Warrenpoint," writes: In reply to queries of "One who Wishes to Know," under heading of "Nurses at Consumption Sanatoria" in your issue of last week, it is most unadvisable for the nurses to use anything belonging to the patients. Here all china cups, plates, glasses, and silver are of a different pattern to prevent any mistake. There are separate washing tubs for the patients and the staff, and all glass-cloths, etc., for patients are cross-barred in red, in contradistinction to blue ones used for the household. "Roughly rinsing" of the patients' utensils, as described, might be a source of danger to the patients themselves. Everything should either be disinfected by boiling in tin baths, or by washing in strong solution of soda and boiling water. We burn the cloths used as dish-cloths for the patients' things. With regard to the second query, I cannot speak from personal experience. Plants do not absorb tubercle bacillus. In many sanatoria the night soil is used in the kitchen garden after being mixed with chlorinated lime and dug into the ground some time before use. Of course, if liquid matter were poured over the growing cabbages many risks, including that of typhoid, might be incurred, as the bacterium coli is contained in the excreta of even healthy persons, but if dug into the ground as a soil fertiliser there can be no danger.

### CIVIL HOSPITALS AND THE ARMY NURSING SERVICE RESERVE.

"RESERVE SISTER" writes: I heartily agree with "Onlooker" in her admiration and appreciation for the good work the Hon. Sydney Holland has done and is always doing on behalf of hospitals and nurses, and in common with all members of my profession are specially grateful to him for his chivalrous defence of nurses against the spiteful attacks made on them by a writer in the "Nineteenth Century" last year. But I fail to see why that fact should preclude me from holding a different view to his on the subject of the A.N.S.R. and the treatment by the civil hospitals of its members. As to the question of gentle birth, it is considered wiser that army sisters ranking with officers should be of their social standing rather than that of the non-commissioned officers and men with whom they have to work. My humble experience certainly bears out the wisdom of this regulation.

"W. S." writes: "Onlooker's" letter in your last issue does not, to my mind, touch the question, viz., Is it fair that those nursing sisters who joined the A.N.S.R. at the commencement of the war without any word of warning that by so doing they would cut themselves off from all future employment in the civil hospitals should be left to "sink or swim" as the case may be? By all means let it be known that in future if they join the A.N.S.R. they do so at their peril, but surely the penalty should not be made retrospective.

"The Hon. Sydney Holland" writes: "W. S." asks me whether I do not think something should be done by the authorities to help members of the A.N.S.R. thrown out of employment by the boycott of civil hospitals. I cannot say because I do not know who the "authorities" are, nor do I believe any boycott exists. I believe the greater part of the A.N.S.R. were nurses doing private work, and to that work they can of course return. She also asks if I think nurses who went to South Africa out of patriotic feelings and who risked their lives for their country should be rewarded by being denied employment. Who denies them employment? If they left their hospitals contrary to the wish of their respective matrons, and without any consideration for the convenience of their hospitals, they can hardly expect to be taken back. If they left private work, as I said before, to private work they can return. If they left with full leave of their matron, as did ours from the London Hospital, they would all be welcomed back as ours have been. But "W. S." would be perhaps surprised to learn how many the excitement of the campaign has unfitted for the comparatively dull routine of hospital life, and my experience is that very few wish to return. As to "patriotic feelings" and "risking of lives" I do not want to be thought to sneer at either, or to say anything unkind. But it was certainly far harder for nurses to make up their minds to stay at home than to do what every nurse from the days of Florence Nightingale has yearned to do, to nurse soldiers during a war. I do not think the nurses who went abroad and have got rewarded and praised for doing very excellent work did any better work than those who unwillingly stayed at home. It is, to my thinking, the nurses who were compelled to stay at home, or who thought it their highest duty to do so, who are the martyrs or heroines, whichever way "W. S." likes to look at it.

---

### STATE REGISTRATION FOR NURSES.

"A Hospital Sister" writes: Miss Helen Todd's rejoinder to my letter on the above subject, is distinctly disappointing, from an advocate of State registration, as she makes no attempt at all to deal with the questions I have raised, *i.e.* (1) What quality and period of training it is proposed to require of candidates for registration. (2) Is any and every "hospital" and "nursing home" to be recognised as a training school, and if not, what is to be the standard? These, to my mind, are not mere questions of detail to be settled by some imaginary "committee," but the crux of the whole matter. Surely it is putting the cart before the horse to agitate for State registration of "nurses" before having a definite understanding of what is to constitute the "nurses" for whom registration is demanded! The minor point as to whether the protection of the public or the nursing profession is the predominant factor in the minds of its advocates is hardly worth pursuing further as it can be only a matter of opinion one way or the other. I do not forget the Queen's nurses, for they prove my argument that very good and valuable work is done without a full hospital training of three years, which I believe is not required of a Queen's nurse, except for a superintendent's post. I fail to see the analogy between a lay helper and one of the useful workers I have spoken of with less than three years' training. My knowledge of "lay helpers" is, I must confess, extremely limited, but I think I am right in saying that they do not wear clerical attire nor call themselves "clergymen." Cottage, district, or parish nurses, whether hospital trained or not, do wear uniform, and are known as "nurses"—the term "sick attendant" is, I fancy, not generally recognised out of an asylum or workhouse. The friendly neighbour says she does a bit of "nursing," and there is no line of demarcation to denote where "looking in on a neighbour" ends and "nursing" begins. It is for the advocates of State Registration to define what they understand by a "nurse" before they demand her registration.

### Appointments.

[No charge is made for announcements under this head, and we are always glad to receive, and publish, appointments. But it is essential that in all cases the school of training should be given.]

---

Acland Home for Paying Patients, Oxford.—Miss Jessie M. Craig has been appointed lady superintendent. She was trained at King's College Hospital, London, and has since been night superintendent at St. Mary's Hospital, Paddington, night superintendent and afterwards sister-in-charge of surgical wards at the Aberdeen Royal Infirmary. She also did duty in South Africa on the Army Nursing Service Reserve for more than two years.

Bute Hospital, Luton.—Miss H. E. Robinson has been appointed matron. She was trained at St. George's Hospital London, and has since held appointments at the East Suffolk Hospital, Ipswich, and the North Devon Infirmary, Barnstaple.

Hull Charterhouse.—Miss Sarah Hutton has been appointed nurse-matron. She was trained at the Children's Hospital, Edinburgh, and the Royal Infirmary, Edinburgh. She has since been nurse at Keighley Infirmary, and Calverley Moor District Hospital, and temporarily in charge of the Evan Fraser Small-pox Hospital at Sutton-on-Hull. She has also had experience in district and private nursing.

Kettering Joint Hospital.—Miss Eliza Weir has been appointed nurse-matron. She was trained at the Belvedere City Hospital, Glasgow, and has since been sister at the Borough Fever Hospital, Bolton.

Memorial Cottage Hospital, Leek.—Miss A. Killey has been appointed staff nurse. She was trained at the Cardiff Infirmary, and has since been engaged in district work.

New Asylum, Tooting Bec, S.W.—Miss Annie Laycock has been appointed assistant matron. She was trained at the Royal Albert Edward Infirmary, Wigan, and has since been charge nurse at the Brook Fever Hospital, and at the National Orthopædic Hospital, Great Portland Street, W. She has also done private nursing.

New Infirmary, Acton Lane, Willesden. — Miss Theresa Francis Jennings has been appointed charge nurse and Miss Jane E. Owen night charge nurse. Miss Jennings was trained at Whitechapel Infirmary and Park Hospital, Hither Green, and was charge nurse under the Metropolitan Asylums Board. Miss Owen was trained at Whitechapel Infirmary and has since been ward sister at the Bethnal Green Infirmary, charge nurse for twelve months under the Metropolitan Asylums Board, and charge nurse at the Victoria Park Hospital.

Shropshire Nursing Federation.—Miss Walsh has been appointed county superintendent. She was trained at the Liverpool Royal Infirmary. For the last two and a half years she has been superintendent of the Newport (Shropshire) Nursing Association and matron of the Nursing Home. She holds the L.O.S. certificate.

Stanley Hospital, Liverpool.—Miss Lily Wright has been appointed sister and Miss Mary Oakes staff nurse. Miss Wright was trained at the Ulster Hospital, Belfast, and has since been staff nurse at the Stanley Hospital, Liverpool. Miss Oakes was trained at the Southwark Infirmary, East Dulwich, London.

West Ham New Infirmary, Leytonstone.—Miss L. Whittington has been appointed matron. She was trained at the Middlesex Hospital, W., and has since been charge nurse at the Hackney Infirmary for five and a-half years, and assistant-matron for two years and eight months. Since April 1902 she has been matron of the Small-pox Hospital at Tottenham. She holds the L.O.S certificate.

WESTMINSTER COTTAGE HOSPITAL, SHAFTESBURY.—Miss Jane Ord has been appointed matron and nurse. She was trained at Guy's Hospital. She has since been sister of the women's ward of the General Hospital, Tunbridge Wells; Matron of the Eye and Ear Hospital, Tunbridge Wells; temporary matron of the Jenny Lind Infirmary for Sick Children, Norwich; and sister of the women's surgical ward, Norfolk and Norwich Hospital.

WORKHOUSE INFIRMARY, CROSS HOUSES, SHREWSBURY.—Miss Elizabeth Mary Parry has been appointed charge nurse. She was trained at the London Hospital and St. Augustine's Nursing Home, Hampstead, and has since been maternity nurse at the latter. She has also been nurse at the North Eastern Hospital, London, and has been employed in private nursing.

### Presentations.

CHELSEA HOSPITAL FOR WOMEN.—The following is a full list of the tokens of esteem presented to Miss Mildred Heather-Bigg on leaving the post of matron of Chelsea Hospital for Women in order to become lady superintendent of Charing Cross Hospital:—From the medical staff, a silver salver and milk jug; the ladies' committee, a handsome armchair, silver hot-water jug, and a hot-water dish (silver); the nurses, a beautiful embossed silver cake-dish with name engraved on it; the servants, an electro and glass biscuit-box and spoon warmer; secretary, a pair of pretty fern pots; assistant secretary, a beautifully bound copy of "Whittier"; resident medical officer, a tastefully bound copy of "Burns"; Dr. and Mrs. Hugh Fenton also presented Miss Heather Bigg with a handsome revolving bookcase.

### "The Hospital" Convalescent Fund.

THE Hon. Sec. acknowledges with many thanks the receipt of 5s. from Nurse Whyte; and 2s. 6d., being a first annual subscription, from Nurse Maud Ruel, who is also thanked for her nice letter.

### Wants and Workers.

NURSE MCALLISTER, 45 West End Lane, Kilburn, N.W., will be glad to supply copies of THE HOSPITAL for the last five years free to any nurse who desires them.

### Death in Our Ranks.

WE are asked to announce the death of Miss Ada Parkinson from enteric fever, contracted when she was nursing a patient. She was trained at Poplar and Stepney Sick Asylum.

### To Nurses.

**WE invite contributions from any of our readers, and shall be glad to pay for "Notes on News from the Nursing World," or for articles describing nursing experiences, or dealing with any nursing question from an original point of view. The minimum payment for contributions is 5s., but we welcome interesting contributions of a column, or a page, in length. It may be added that notices of appointments, entertainments, presentations, and deaths are not paid for, but that we are always glad to receive them. All rejected manuscripts are returned in due course, and all payments for manuscripts used are made as early as possible after the beginning of each quarter.**

### For Reading to the Sick.

"WHOSE SERVICE IS PERFECT FREEDOM."

WHAT asks our Father of His children save
Justice and mercy and humility,
A reasonable service of good deeds,
Pure living, tenderness to human needs,
Reverence, and trust, and prayer for light to see
The Master's footprints in our daily ways?

*Whittier.*

Thou that hast given so much to me,
Give one thing more, a grateful heart
Not thankful when it pleaseth me,
As if Thy blessings had spare days;
But such a heart, whose pulse may be
Thy praise.

*G. Herbert.*

What is meant by our neighbour we cannot doubt; it is everyone with whom we are brought into contact. First of all, he is literally our neighbour who is next to us in our own family and household; husband to wife, wife to husband, parent to child, brother to sister, master to servant, servant to master. Then it is he who is close to us in our own neighbourhood, in our own town, in our own parish, in our own street. With these all true charity begins. To love and be kind to these is the very beginning of all true religion. But, besides these, as our Lord teaches, it is everyone who is thrown across our path by the changes and chances of life; he or she, whosoever it be, whom we have any means of helping—the unfortunate stranger whom we may meet in travelling, the deserted friend whom no one else cares to look after.—*A. B. Stanley.*

Do right, and God's recompense to you will be the power of doing more right. Give, and God's reward to you will be the spirit of giving more: a blessed spirit, for it is the Spirit of God himself, whose Life is the blessedness of giving. Love, and God will pay you with the capacity of more love; for love is Heaven—love is God within you.

*F. W. Robertson.*

For patience, when the rough winds blow!
For patience, when our hopes are fading—
When visible things all backward go,
And nowhere seems the power of aiding!
God still enfolds thee with His viewless hand,
And leads thee surely to the Fatherland.

*From the German.*

We have only to be patient, to pray, and to do His will, according to our present light and strength, and the growth of the soul will go on. The plant grows in the mist and under clouds as truly as under sunshine. So does the heavenly principle within.—*W. E. Channing.*

There is a blessed peace in looking for nothing but our daily task, and our portion of Christ's cross, between this day and the appointed time when we shall fall asleep in Him.—*Wilberforce.*

### Echoes from the Outside World.

### Movements of Royalty.

ON Sunday the King, accompanied by the Queen and Princess Victoria, attended divine service in the Private Chapel at Windsor Castle. Amongst the congregation was the Home Secretary, who had come over-night to the Castle to present the new Primate to King Edward. The Archbishop of Canterbury preached the sermon and read the lessons.

The King, who has quite recovered from the mild attack of influenza from which he suffered last week, returned to London, accompanied by the Queen, on Monday. The public assembled in large numbers to greet his Majesty along the route from Paddington to Buckingham Palace, and by the heartiness of their demonstration, endeavoured to prove their satisfaction that the Sovereign's health was restored. At the opening of Parliament on Tuesday, the State Coach will be used, and will be drawn by eight cream horses, the other four carriages having each six black steeds.

### Mr. Chamberlain's Tour in South Africa.

THE experiences of Mr. Chamberlain in Bloemfontein have been of a somewhat mixed character. At Government House there was a smoking concert attended by all the chief men in the Orange River Colony. Mr. Chamberlain held long conversations with some of the guests. An amusing incident occurred in the course of a conjuring performance. The conjurer had two members of the audience on the platform in order to assist him in performing a trick; and after accomplishing an apparently impossible feat, he astonished the company by informing his two coadjutors that they were lacking in nerve. The coadjutors happened to be Messrs. Manie Botha and Wessels, two of the best known of Boer Commandants, and the conjurer's remark naturally excited roars of laughter. Mr. Chamberlain's more serious work consisted in receiving deputations, headed by Christian De Wet and Judge Herzog. In the course of the proceedings the Colonial Secretary had to rebuke the delegate for presenting an address which impugned his good faith and that of the British Government. More pleasant was the presentation of an address from the loyal burghers who fought for the British during the war, and the garden party in his honour which was attended by all the prominent residents. On Saturday evening the Colonial Secretary was entertained at a banquet, and in the course of a powerful speech, he urged the Boers to abandon their dissensions. He left by train for Grahamstown on Monday amid a hearty popular demonstration, and on his arrival in the Settlers' Town delivered a striking address.

### Vandalism at Hampton Court.

FRESH injury has been done to some of the valuables at Hampton Court. A few months ago it was noticed that the scroll work attached to a handsome brass lock in one of the State apartments—open to the public—had been torn away. Towards the end of December two beautiful paintings, one by Holbein and the other by Kneller, were found to have been damaged. This time it is neither brasswork nor pictures which have attracted the attention of the despoilers, but some of the valuable tapestry. The centre one of three Flemish pieces, which hang in the room known as the "horn room," has received a cut with some sharp instrument. The cut is semi-circular in shape, and is about six inches long. The tapestries are enclosed within an old carved oak railing, and opposite is a staircase by which dishes were formerly brought from the Royal kitchens to the great hall. The "horn room" was so called, because for many years a large collection of horns, made by Queen Elizabeth, were accommodated there, but of late most have been removed to the great hall. At present no trace of the culprits has been found.

### "Edna Lyall."

MISS ADA ELLEN BAYLY, well known to the reading public by her *nom-de-plume* of "Edna Lyall," died at Eastbourne on Sunday. She served a long apprenticeship to literature, having spent five years in vainly endeavouring to persuade publishers to accept her novels. Her first work, "Won by Waiting" failed to make any mark, but "Donovan" was more warmly received, and owing to the sensation made at the time of its publication by the avowed Atheism of Mr. Bradlaugh, whom the hero was supposed to resemble, it was much discussed. "We Two" turned the tide in Miss Bayly's favour, and all her other works have been more or less popular. Though always delicate, Miss Bayly for years held a Sunday afternoon class in Eastbourne, where she lived, for young shop assistants. Out of the proceeds of her writings she presented the Church of St. Saviour, Eastbourne, with three bells, named "Donovan," "Erica," and "Hugo," after three characters in her novels.

### Society of Women Artists.

THERE are several pictures of sufficient merit in the 48th Exhibition of the Society of Women Artists, just opened in Suffolk Street, to repay a visit, although it must be admitted that some of the contributions are by no means of a high enough order to warrant their acceptance or the position which they occupy. The water colours are the most deserving of praise. A study of a woman at prayer by Miss Vera Hubbard, should be noticed, as well as "Little Bo-Peep," a charming drawing by Miss Janet Fisher. Mrs. A. Martin's "An Eastern Enchantress" is sure to attract attention, the painting of the golden drapery and of the scarf above the woman's head being particularly clever. Two good portraits of dogs, one a Newfoundland, the other a Japanese, by Miss F. C. Fourman, will enhance that lady's reputation as a dog-artist, and Miss Beatrice Bright contributes not only a seascape and a landscape but a well-modelled portrait of a girl in Italian dress. The two small rooms at the end of the gallery have examples of arts and crafts, all wrought by women. Some of the work is exceedingly good, the bookbindings are especially commendable, and so are the embroideries.

### "The Light that Failed."

AN adaptation of Mr. Rudyard Kipling's book, "The Light that Failed," dramatised by "George Fleming" (Miss Constance Fletcher), was produced on Saturday at the Lyric Theatre. The first scene is laid in the Soudan, and there the War Correspondents meet and talk and chaff, and, as it were, prepare the listener for the blindness which afterwards overtakes Dick Helder. In the next scene Maisie appears at work in her studio with the Red-haired Girl, and there Helder introduces to her notice the wretched gutter snippet Bessie Broke. Later, Maisie, placing Art before Love, refuses Helder when he asks her to become his wife. In the next act the picture "Melancholia," for which Bessie has acted as model, is nearly completed, and though it has been frequently painted when the artist has been under the influence of drink, it is a grand piece of work. Helder discovers Bessie has been trying to ensnare his friend Torpenhow, and turns her away. Then, in revenge, Bessie maliciously cuts the picture, and Helder is suddenly smitten with blindness. In the final act, though nothing can restore the artist's picture nor his eyesight, he obtains an unlooked-for reward. Mr. Forbes Robertson as the hero has a character admirably suited to him; Miss Gertrude Elliott is tender and graceful as the heroine, and a remarkable success was made by Miss Nina Boucicault as Bessie, who grasped the innate vulgarity of the character in a masterly manner.

### Notes and Queries.

**The Editor is always willing to answer in this column, without any fee, all reasonable questions, as soon as possible.**

**But the following rules must be carefully observed:—**

**1. Every communication must be accompanied by the name and address of the writer.**

**2. The question must always bear upon nursing, directly or indirectly.**

**If an answer is required by letter a fee of half-a-crown must be enclosed with the note containing the inquiry, and we cannot undertake to forward letters addressed to correspondents making inquiries. It is therefore requested that our readers will not enclose either a stamp or a stamped envelope.**

*Massage.*

(141) 1. Is massage much practised in England? 2. Is there any means by which I could get introductions to the medical staff of a hospital or convalescent home? 3. Would my certificate, obtained in London, be acknowledged abroad?—*A. H. R.*

1. Yes. 2. Only by personal introduction either through friends or by coming under their notice in a nurse's capacity. 3. Much depends upon the certificate.

1. Is there any hospital in London which takes pupils in massage? 2. Are there good openings for certificated masseuses either privately or by becoming attached to a staff of a nursing home?—*G. A. N.*

1. The National Hospital for the Paralysed and Epileptic, Queen's Square, Bloomsbury, W.C., takes pupils for massage. 2. See replies to other correspondents under this heading. There is always a difficulty at first in working up a connection.

I desire very much to obtain a certificate in massage, will you kindly inform me if I can do so in Brighton?—*Anxious.*

There is no public school of massage in Brighton, but you will doubtless be able to get instruction and a certificate privately. You can obtain a list of qualified teachers from the Incorporated Society of Trained Masseuses, 12 Buckingham Street, Strand, W.C.

*Lectures.*

(142) Will you kindly tell me what is the proper fee to charge for giving a short course of lectures on sick nursing? Is there any particular book you would recommend as a help in preparing for them?—*District Nurse.*

The usual fee would be 10s. a lecture. Write to the Secretary, the National Health Society, 53 Berners Street, London, W., as to literature on the subject.

*Etiquette.*

(143) Will you kindly tell me if there is an inexpensive book on etiquette for nurses and probationers?—*J. D.*

Perhaps some of our readers can answer this question.

*Training School.*

(144) What makes a hospital a "recognised training school for nurses," and qualifies it to give certificates to nurses after three years' work therein?—*L. T.*

You can obtain full information on this point from the Local Government Board, Whitehall, S.W. The Board recognises the training of nurses in certain hospitals which provide an approved course of instruction, and maintain a resident medical officer as well as a fully qualified superintendent nurse. There is no official list published of training schools.

*Home.*

(145) I purpose opening a home for maternity patients, will you kindly tell me if I require a license, or to be in any way registered?—*F. S. C.*

No. But if you intend to act as a midwife you must conform to the provisions of the Midwives Act.

Can anyone recommend a home in the country where a lady suffering from loss of memory could be received at moderate terms?—*N. W.*

There is a long list of homes for women of all ages and requirements in the "Englishwoman's Year-Book," published by Messrs. Adam and Charles Black.

Will you kindly tell me how a young man of twenty-one, suffering from tuberculosis and scrofula, could obtain admission into a home for incurables. His friends are very poor. Does it need influence or votes? Where could I get particulars?—*Matron.*

We fear he is not eligible for most of the incurable homes, which are entered by votes. You might write and state his case to the Hospital of St. John and St. Elizabeth, Grove End Road, St. John's Wood, London, N.W. Will not the guardians of his parish contribute to his support?

Can you tell me the names of any homes for epileptic and insane children of the middle and upper classes?—*F. B.*

You will find a list of licensed homes in "Burdett's Hospitals and Charities." The Holloway Sanatorium for the Insane, St. Ann's Heath, Virginia Water, is open to all cases of mental weakness amongst the upper and middle classes, and the admission is by payment.

*Nursing Home.*

(146) I have received many letters from patients who would like to come to me, mostly from London addresses, but, on finding that I live in a provincial town, they express their regret and decline. Would it benefit me if I removed to the suburbs of London? I do not care to risk the expense unless I thought it would be an advantage.—*A. N. S.*

This is a case on which we can offer no opinion. Competition in nursing homes is very keen in London, and therefore patients ought to be secured, and expenses calculated, before any decisive step is taken.

*Hospital Training.*

(147) Will you kindly tell me if training at any one of the London hospitals is better than infirmary training, and if the certificate is as valuable?—*H. R.*

There is no more valuable certificate than that given for training at the best London hospitals.

I am axious to enter a hospital for training; I am willing to give my services, but I should like to know if my age, 36, is against me.—*K. H.*

Thirty-five is the usual age-limit for probationers, you would therefore have great difficulty in obtaining training. Your best chance would be at a workhouse infirmary.

*District Training.*

(148) Will you kindly tell me where to apply for information regarding a six months' course of training in district work? Is a premium required, and is it possible to obtain district training with less than one year's previous general training?—*E. E. S.*

Two years' general training is required by the Queen Victoria's Jubilee Institute for Nurses (apply to the General Superintendent, St. Katharine's Precincts, Regent's Park, N.W., for all particulars), but the Affiliated Benefit Nursing Associations, 12 Buckingham Palace Road, S.W., trains for what are known as cottage nurses, and you might write to them. Also the Maternity Charity and District Nurses' Home, Howards Road, Plaistow, E., might help you.

*Abroad.*

(149) Will you kindly give me the address of an English private nursing institute at Brussels or Mentone?—*Anxious Inquirer.*

The Nice Nursing Institute, Villa Pilatte, Avenue Désambrois, Nice, is the nearest to Mentone. We have no information as to private establishments in Brussels.

*Elastic Bandages.*

(150) Will you kindly tell me of any way in which elastic bandages may be cleaned? I am often asked this by persons whose bandages are a great expense to them.—*Mary.*

Lay the bandage on a board and brush well with good soap lather, made from "Sunlight" or "Fels-Naphtha" soaps for this. Let the bandage lie for one or a few hours, and then rinse carefully in several cold waters, drying it slowly in a cool shady place. Do not wring the bandage, but squeeze out the moisture by folding in a towel and pressing it between the hands.

*Lodging.*

(151) Can you give me some idea of what a village district nurse usually pays for lodging?—*A. D.*

This would depend on the social status of the nurse, and also on the locality of the village. The Secretary, the Affiliated Benefit Nursing Associations, 12 Buckingham Palace Road, S.W., would be able to advise you.

**Standard Books of Reference.**

**"The Nursing Profession: How and Where to Train." 2s. net; post free 2s. 4d.**

**"Burdett's Official Nursing Directory." 3s. net; post free, 3s. 4d.**

**"Burdett's Hospitals and Charities." 5s.**

**"Hospital Expenditure: The Commissariat." 2s. 6d.**

**"The Nurses' Dictionary of Medical Terms." Cloth, 2s.; leather, 2s. 6d. net.; post free, 2s. 8d.**

**"Burdett's Series of Nursing Text-Books." 1s. each.**

**"A Handbook for Nurses." (Illustrated). 5s.**

**"The Physiological Feeding of Infants." 1s.**

**"The Physiological Nursery Chart." 1s.; post free, 1s. 3d.**

**All these are published by the SCIENTIFIC PRESS, Ltd., and may be obtained through any bookseller or direct from the publishers, 28 and 29 Southampton Street, London, W.C.**

### Travel Notes.

**By Our Travelling Correspondent.**

### CXVII.—HAUNTED SAN MARCO.

The convent of San Marco stands in a somewhat uninteresting square, but its walls, could they speak, would tell such thrilling stories as would draw all men thither. Even now it is one of the most visited spots in Florence. Its halls, corridors, and cells are haunted; here lived, struggled, sinned, suffered, and triumphed, the great Savonarola, one of the most striking figures of mediæval times; here wandered with him the gentle, imaginative Fra Silvestro, who like St. Peter fell away in time of dire temptation, but nevertheless eventually showed the triumph of spirit over body in the last dread moment. Here, too, lived the indomitable Fra Domenico, whose spirit nothing could quench—a man evidently of iron nerve, and probably not of strong imagination—who at the moment of execution was with difficulty restrained from singing the Te Deum aloud, such was his state of spiritual exaltation. Here, too, Baccio della Porta ended his days; he is better known to us as the Fra Bartolommeo, the faithful friend and follower of Savonarola; and here, too, worked and prayed that pure, religious, and artistic soul and genius, Fra Angelico.

### "Savonarola's Soul went out in Fire."

The above line of Mrs. E. Barrett Browning's so exactly meets our ideas of the martyr's death, that in thinking of that event we almost unconsciously think of her words.

The great religious reformer was born in 1452, and early became a Dominican monk; there is a tradition, but for the truth of it I cannot vouch, that his entering the cloister was in consequence of an unhappy attachment to a lady of the powerful Strozzi family. I have often looked up at the grim walls of the Palazzo and wondered whether the young Girolamo looked longingly at their strength and majesty, and yearned for his love within. It is quite possible that the story is an invention, but it is easy enough to suppose it true, for the strong, harsh features of the mighty Frate seem to speak of a man of ardent temperament and strong passions; though both were purified by burning faith, there seems something in the expression which tells of a continued warfare between the flesh and the spirit, though the latter triumphed through his reliance on his God, for whom he gave his life. Probably he was not a very agreeable man, he must have been too rugged in manner, and too independent in spirit to be a favourite with those in high places, but undoubtedly he inspired burning zeal and deep affection in those who came under his influence. There was much, too, of the fanatic about him, that wholesale sweeping away of everything that savoured of this world, its pleasures and vanities.

### The Auto-da-Fé.

Villari's life of the Frate is delightful reading, and we have much interesting matter relating to him in Harford's "Life of Michelangelo.

"A pyramidal scaffold was erected opposite the palace of the signory. At its base were to be seen false beards and hair, masquerading dresses, cards and dice, mirrors and perfumery, beads and trinkets of various sorts; higher up were arranged books and drawings, busts and portraits of the most celebrated Florentine beauties; and even pictures by great artists, condemned on very insufficient grounds as indecorous or irreligious. Even Fra Bartolommeo was so carried away by the enthusiasm of the moment as to bring his life Academy studies to be consumed on this pyre, forgetful that, in the absence of such studies, he could never himself have risen above low mediocrity." Savonarola was a great reformer, a fervent Christian, a devout Catholic, one who willed only good to all mankind and thought nothing of himself, but he was an impossible kind of man, and with all his devotion and self-sacrifice accomplished little or nothing, because he did not understand human nature.

### The Beginning of the End.

Unfortunately he had raised the ire of the Franciscans, always rivals of the Dominicans. After the failure of the ordeal by fire in the Piazza, the Franciscans and the fickle mob rushed to San Marco, and a free fight took place in the church and convent. Some of the San Marco monks forgetting the rule of love and forgivenness of injuries, replied to the rabble with musket shots and even beat their assailants with the bronze altar ornaments. According to Villari, Savonarola gave his last address to his followers in the once peaceful library. "My sons," he said "in the presence of God, standing before the sacred host and with my enemies already in the convent I now confirm my doctrine. What I have said came to me from God and He is my witness in Heaven that what I say is true."

After this he was taken to the Bargello and there cruelly tortured with a view to compelling him to deny the Divine inspiration. At one moment frail nature trembles and all but recants, but the God in whom he trusted stands by him and he rises again above the temptation. From there, separated from his companions until their last night on earth, he was taken to a cell high up in a tower of the Palazzo Vecchio where he remained a month.

### His Last Words.

These have become historical. When he was stripped of his habit, the Bishop of Vasona said in a trembling voice. "I separate thee from the church militant and triumphant." The victim replied in an unmoved voice, "Militant, not triumphant—your church is not triumphant!" As the chain was being fastened round his body a priest asked him, "In what frame of mind do you endure this martyrdom?" His answer was, "The Lord has suffered as much for me!"

So died that singular and godly man, mistaken perhaps, and not free from earthly pride and ambition, but a bright and shining light in an age given up to much cruelty, lust, avarice, and tyranny.

### TRAVEL NOTES AND QUERIES.

To Holiday Makers in May and June.—Any nurses or other hard workers who would like sixteen days abroad, from May 23rd to June 7th, may be glad to hear that there is such a trip organised to see the Italian lakes, returning via Verona and Venice The cost will be 13 guineas. Anyone wishing to hear more can do so by sending me a stamped addressed envelope. It is a private affair, in no way undertaken to make money, but simply to provide by means of co-operation a pleasant holiday to the organiser and those who go with him. I have sent several friends on former occasions, and it has always been an unqualified success. Last year the trip was to Rome via the Riviera, and the year before to Florence and Venice. The list is made up very early, so that anyone thinking of joining should let me know without delay.

**Rules in Regard to Correspondence for this Section.—All questioners must use a pseudonym for publication, but the communication must also bear the writer's own name and address as well, which will be regarded as confidential. All such communications to be addressed "Travel Editor, 'Nursing Section of *The Hospital*,' 28 Southampton Street, Strand." No charge will be made for inserting and answering questions in the inquiry column, and all will be answered in rotation as space permits. If an answer by letter is required, a stamped and addressed envelope must be enclosed, together with 2s. 6d., which fee will be devoted to the objects of "The Hospital" Convalescent Fund. Any inquiries reaching the office after Monday cannot be answered in "The Hospital" of the current week.**

